# A Case Report on Contralateral Transient Diplopia After Regional Dental Anaesthesia: Do Anatomical Variations Play a Key Role?

**DOI:** 10.7759/cureus.31629

**Published:** 2022-11-18

**Authors:** Constantina A Tasioudi, Dimosthenis Chrysikos, Philippos Tasioudis, Theano Demesticha, Theodore Troupis

**Affiliations:** 1 Dentistry, Aristotle University of Thessaloniki, Thessaloniki, GRC; 2 Dentistry, Korgialeneio-Benakeio, General Hospital of Athens, Athens, GRC; 3 Anatomy, National and Kapodistrian University of Athens, Athens, GRC; 4 Medicine, Aristotle University of Thessaloniki, Thessaloniki, GRC; 5 Surgery, Medical University of Athens, Athens, GRC

**Keywords:** dental anatomy, clinical dentistry, anatomical variations, complications, local dental anaesthesia

## Abstract

One of the most common procedures in everyday dental surgical practice is the inferior alveolar nerve block anesthesia. The procedure is safe, though various complications may arise. Among them, ophthalmological complications such as temporary loss of vision, amaurosis, diplopia, or ophthalmoplegia are very rare, although they do occur.

This case report highlights an inadvertent complication of contralateral temporary diplopia after inferior alveolar nerve block anesthesia that was administrated in a patient who was set to undergo root canal treatment. Anatomical variations of the middle meningeal arteries and maxillary arteries or the sympathetic vasoconstrictor nerve (carrying important sympathetic fibers) along with intravascular administration of the anesthetic may cause uncommon ophthalmological complications such as transient double vision.

## Introduction

Regional dental anesthesia, undoubtedly, is the first and most important procedure in dental practice as a pain relief method. Although several techniques have been described to achieve an optimal anesthetic result, a dentist should always remember that the anatomical area where the anesthesia is administrated may have anatomical variations that can compromise the result. Extensive knowledge of the anatomical variations in this region is of paramount importance not only for adequate local anesthesia but equally for dental and maxillofacial operations.

The anatomic space where this procedure occurs, the pterygomandibular space (PM), is at high risk because of its neurovascular contents such as the lingual nerve (LN) and the inferior alveolar neurovascular structures, and their associated branches such as the elements of the mylohyoid neurovascular bundle.

Anson (1966) and Clemente (1985) have described an artery called the lingual branch that comes off the inferior alveolar artery (IAA) near its origin and descends with the LN to supply sensation (both gustatory (taste) and non-gustatory) to the anterior two-thirds of the tongue [1]. Ferner (1963) illustrates an *N. lingualis cum arteria comit*., which is shown to branch off a posterior deep temporal artery (in the deep route of the maxillary artery) and descend vertically in the PM space [1]. Baumel et al. (1961) state that the accessory meningeal artery (AMA) arises from the deep route of the maxillary artery (MA) and will supply small branches to the infratemporal fossa, including the medial and lateral pterygoid muscles and the lingual and inferior alveolar nerves [1]. This lingual branch or artery to the lingual nerve (ALN) must traverse the PM space, thus placing this region at risk during any procedure [1].

This case report aims to shed light on a rare complication of temporary controlateral diplopia after inferior alveolar nerve block anesthesia that was administrated in a young female patient who was scheduled for a root canal treatment. Possible correlations of this inadvertent complication of dental anesthesia are discussed along with anatomical variations that may compromise the optimal anesthetic result.

## Case presentation

**Figure 1:**
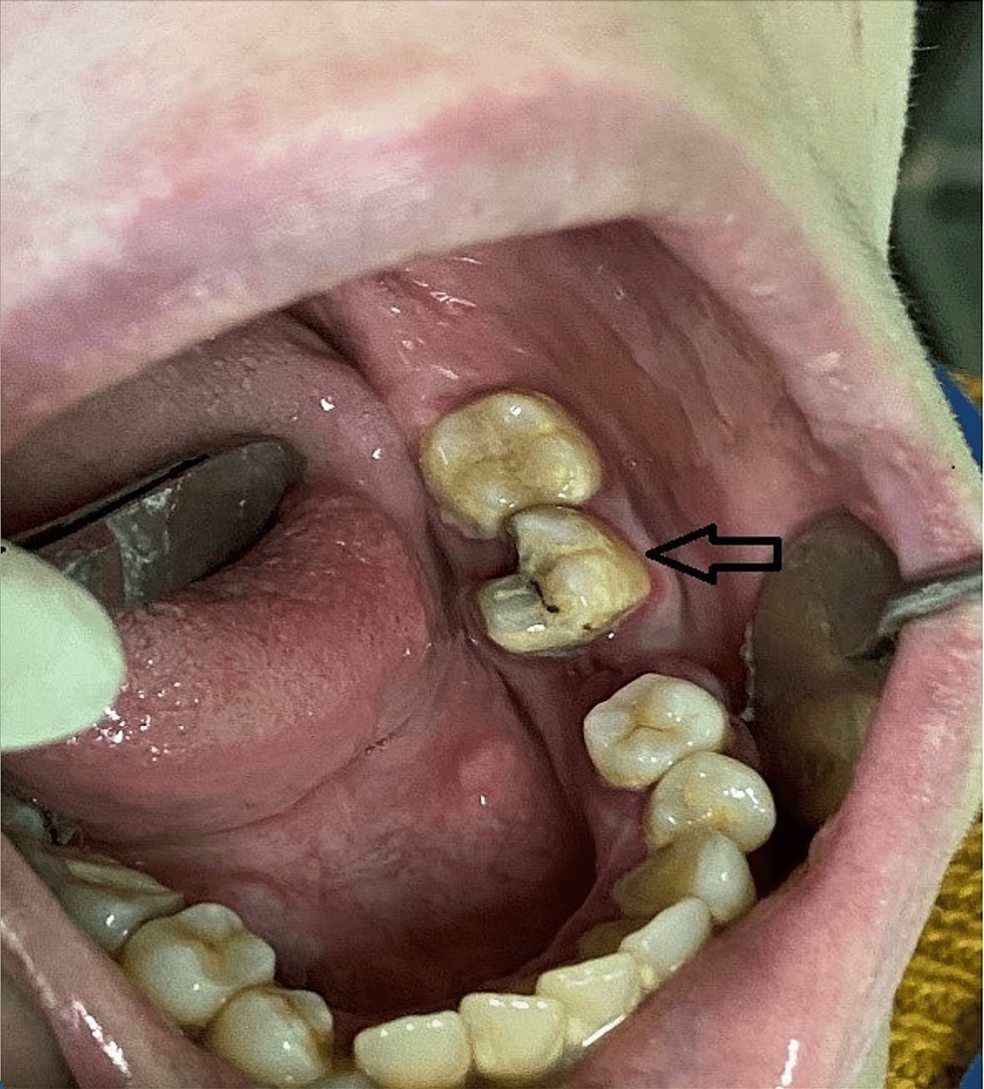
Second left lower molar diagnosed with acute irreversible pulpitis

An 18-year-old female patient came to the General Hospital of Athens Korgialeneio-Benakeio, Greece, with principal symptomatology of pain in the left lower molar region. After clinical and X-ray examination, the patient was diagnosed as having acute irreversible pulpitis in tooth 37 (Fédération Dentaire Internationale (FDI) dental numbering system)/lower left permanent second molar (Figure 1). Root canal treatment of the same tooth was planned under local anesthesia with articaine 4% with epinephrine 1:100000.

The medical history of the patient was unremarkable and no previous dental treatment was referred until then. Before administrating the anesthetic agent, the young patient’s systolic and diastolic blood pressure, and heart rate were assessed and were within the normal range (101 mmHg systolic and 73 mmHg diastolic arterial blood pressure, heart rate at 73 pulses per minute).

Inferior alveolar nerve block anesthesia was given to the patient’s left side unilaterally, using an extra-long (30 mm) 27-gauge needle. Aspiration was performed and was negative. Two minutes after injecting 1.7 ml of 4% articaine with epinephrine, 1:100000 (4% Articamine), the patient complained of compromised vision in her right eye. For the next 20 minutes, the patient complained of double vision in her right eye even though the vision in her left eye was unaffected. Diplopia has been referred to in several articles as the main transient ophthalmological complication due to regional dental anesthesia but not to the contralateral side of the face [2-6].

**Figure 2:**
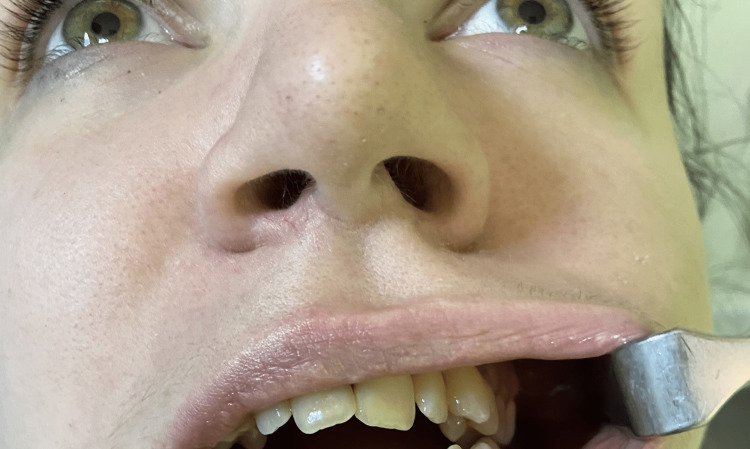
The patient was asked to move her eyes following an object in order to check their motility

A motility test was performed by giving instructions to the patient to move her eyes in all directions, which indicated normal ocular movement of both eyes (Figure 2). When the patient was asked to read a chart with one eye at a time, it was found that there was a double vision in her right eye and normal vision on the left side. The facial muscle movement was normal, which indicated that the facial nerve function was unaffected, and no blanching or burning sensation of oral mucosa was noticed on either side. This reaction, to our knowledge, has not been previously reported.

The condition was diagnosed as temporary diplopia during inferior alveolar nerve block anesthesia. The double vision disappeared within 30 minutes after the initial symptom onset and the patient’s eyesight returned to normal. The patient was followed up for one hour after the appointment was completed as well as the day after when she came back to culminate the therapy. Her right eye vision was intact.

A similar case report was published by Barodiya et al. in 2017 [7]. However, in that particular case report, the patient presented temporary blindness ipsilaterally to the anesthetic agent’s side of the administration, whereas in our case the complication occurred contralaterally. 

## Discussion

Ophthalmological complications

There have been several reports of ocular complications following the administration of local dental anesthesia [2]. Symptoms and signs which include amaurosis, diplopia, miosis, mydriasis, palpebral ptosis, enophthalmos, facial blanching, hematoma formation, and even permanent blindness have been reported [2].

Double vision after administration of regional anesthesia for dental and maxillofacial operations is an unusual complication, that occurs with a frequency of 39.8% among all ophthalmologic complications according to table III of the analysis by von Arx et al. [4]. The cause is not clear but the most common theory is back pressure from an inferior alveolar nerve block traveling back into the maxillary artery via retrograde flow and gaining access into the middle meningeal artery to the ophthalmic artery that causes symptoms such as diplopia to appear [2,5]. The maxillary artery according to Meyer travels lateral to the lingual nerve and the inferior alveolar nerve, or medial to both; the maxillary artery is located lateral to the lingual nerve and medial to the inferior alveolar nerve, or medial to the lingual nerve and lateral to the inferior alveolar nerve [8]. Furthermore, in 4% of patients, the ophthalmic artery is established not from the internal carotid artery but from the middle meningeal artery, which follows an uninterrupted flow from the external carotid artery [9]. Petrelli et al. surmised that the anesthetic solution can enter the orbit directly through a bony defect in the wall of the maxillary sinus [10].

Kronman et al. proposed that in perivascular trauma, the intra-arterial injection stimulates the sympathetic fibers running alongside the internal maxillary artery until it reaches the orbit, which would account for vasoconstriction and mydriasis [2,11]. There is also a local diffusion phenomenon communicating with the pterygoid venous plexus and the ophthalmic vein through the orbital fissure [2,12,13]. Goldenberg et al. also reported that local anesthetic could pass through the cavernous sinus via the route of the ophthalmic vein, ultimately reaching the ophthalmic artery through a wide range of anastomoses [2,14].

These symptoms usually resolve with time, lasting from a few minutes to a few hours [15]. When ocular complications persist, an ophthalmology consultation is of paramount importance. Aspiration at the time of administration of local anesthesia is very important and minimizes the risk of ocular complications [2].

## Conclusions

Regional anesthesia is a routine technique in all oral and maxillofacial practices. The anesthetic agents that are administrated are efficient and safe but do carry implicit risks. Many of the ocular complications, such as accommodation disturbance, amaurosis, enophthalmos, miosis, mydriasis, ophthalmoplegia, ptosis, or diplopia (as discussed in this case report) that can be either permanent or temporary, are rare but do occur. Minimizing adverse outcomes might be achieved by using the proper regional anesthetic agent in situations such as patient allergies, pregnancy, medical history, and estimating dosages to avoid toxicity, and aspirating while administrating regional anesthesia to help dissuade systematic and/or transient complications. Such techniques are essential for every dentist to minimize undesired side effects when giving regional anesthesia.

However, the role of anatomical variations of the inferior alveolar nerve, maxillary nerve, and mental and lingual nerve should never be neglected and may correlate to inadvertent complications such as ophthalmological ones. Preoperative knowledge of anatomical variations is essential in order not to misdiagnose and to guide proper surgical decision-making, which in turn will lead to optimal patient care.
